# Der Wohlfahrtsstaat als politische Quelle sozialer Solidarität. Eine institutionentheoretische Perspektive

**DOI:** 10.1007/s11614-023-00527-1

**Published:** 2023-05-15

**Authors:** Stefanie Börner

**Affiliations:** Institut für Gesellschaftswissenschaften, Otto-von-Guericke-Universität Magdeburg, Universitätsgebäude 40, Zschokkestraße 32, 39104 Magdeburg, Deutschland

**Keywords:** Ideen, Institutionen, Institutionentheorie, Moderne Wohlfahrtsstaaten, Organisationen, Soziologie des Sozialstaates, Ideas, Institutions, Institutionalist theory, Modern welfare states, Organisations, Welfare state theory

## Abstract

Der Artikel untersucht den Wohlfahrtsstaat als politische Quelle von Solidarität und fragt nach den Solidarisierungspotentialen von Sozialpolitik. Können derart abstrakte, staatlich organisierte und über Beitrags- und Steuerzahlungen formalisierte Unterstützungsbeziehungen in einer Gesellschaft überhaupt Solidarität hervorbringen und wenn ja, welche Mechanismen sind dafür verantwortlich? Die Analyse beruht auf der Annahme, dass soziale Solidarität in hochgradig differenzierten Gesellschaften auf politische Steuerungsleistungen angewiesen ist, und betrachtet Sozialpolitik als eine wesentliche Voraussetzung für die Entstehung von Solidarität unter Fremden. In der Tradition der klassischen Soziologie wird Solidarität hier als dynamisches und elastisches Konzept gedacht (Abschn. 2). Daran anknüpfend untersucht der Beitrag die Solidarisierungspotentiale moderner Wohlfahrtspolitik aus institutionalistischer Perspektive. Abschn. 3 stellt die zentralen institutionentheoretischen Annahmen vor und arbeitet drei solidaritätsrelevante Wirkmechanismen heraus: die Kompassfunktion (normative Ebene), die Stabilisierungsfunktion (interpersonelle Ebene) und die Scharnierfunktion (organisationale Ebene). Aus institutionentheoretischer Sicht strukturieren sozialpolitische Institutionen in ihrem dynamischen Zusammenspiel aus Leitideen, Institutionen und Organisationen das Bewusstsein und Verhalten und prägen mittel- und langfristig deren individuelle Präferenzen und Einstellungen (bspw. in Bezug auf Umverteilungspräferenzen oder Gerechtigkeitsvorstellungen). Entsprechend stellt der Wohlfahrtsstaat einen wesentlichen Produktions- und Reproduktionsfaktor von Werten, Handlungspraktiken und horizontalen wie vertikalen gesellschaftlichen Beziehungen dar. Das Theoriemodell wird anschließend anhand der Sozialgeschichte und Funktionsweise des deutschen Sozialstaates beispielhaft angewendet, obgleich nicht in einem hypothesentestenden, sondern empirisch plausibilisierenden Sinn unter Zuhilfenahme der soziologischen und sozialhistorischen Wohlfahrtsstaatsforschung. Die Analyse erlaubt es, ambivalente bis krisenhafte Sozialstaatsdeutungen und widersprüchliche gesamtgesellschaftliche Zeitdiagnosen wie Polarisierung vs. neue Solidaritäten besser einordnen und aufeinander beziehen zu können.

## Abstract (en)

The article examines the welfare state as a political source of solidarity and seeks to investigate the solidarisation potential of social policy. Can such abstract, state-organised relationships of mutual support, formalised through contributions and tax payments, generate solidarity in a society at all, and if so, what mechanisms are responsible for this? The analysis is based on the assumption that social solidarity in highly differentiated societies depends on political management and thus considers social policy as an essential precondition for the emergence of solidarity among strangers. In the tradition of classical sociology, solidarity is conceived as a dynamic and elastic concept (Sect. 2). Following on from this, the article examines the solidarisation potentials of modern welfare policy from an institutionalist theory perspective. Sect. 3 presents the central institutionalist assumptions and elaborates three mechanisms of action relevant to solidarity: the compass function (normative level), the stabilisation function (interpersonal level) and the hinge function (organisational level). From an institutional theory perspective, welfare state institutions in their dynamic interplay of guiding ideas, institutions and organisations structure the consciousness and behaviour of actors and shape their individual preferences and attitudes (e.g. with regard to redistribution preferences or ideas of justice) in the medium and long term. Accordingly, the welfare state represents an essential production and reproduction factor of values, action practices and horizontal and vertical social relations. Drawing on sociological and socio-historical welfare state research, the theory model is then exemplarily applied to the social history and functioning of the German welfare state, although not in a hypothesis-testing but empirically plausibilising sense. The analysis makes it possible to better classify and relate the ambivalent and crisis-ridden interpretations of the welfare state and contradictory contemporary diagnoses of society as a whole, such as polarisation vs. new solidarities.

## Einleitung

Die sozialpolitischen Krisenantworten auf die Covid-19-Pandemie haben die gesamtgesellschaftliche Bedeutung des öffentlichen Sektors und eines „starken Sozialstaat[es]“^1^ auf eine Weise verdeutlicht, wie es die letzten 30 Jahre der Wohlfahrtsstaatsdebatte nicht vermochten. Zugleich haben Diskussionen wie die über die Angemessenheit der Infektionsschutzmaßnahmen und eine zeitlich befristete Impfpflicht gegen das Virus die Diskussion um das adäquate Ausmaß wohlfahrtsstaatlicher Intervention erneut entfacht. Nicht nur, ob Impfen ein Akt der Solidarität sei, wird kontrovers diskutiert, sondern auch, welche politischen Instrumente zum Schutz vor einer Infektion und zur Überbrückung pandemiebedingter Engpässe angebracht seien. Auch wenn die Argumentationsfiguren keineswegs neu sind, wirft die Debatte doch die Frage nach den Grenzen und der Bedeutung des Wohlfahrtsstaates mit neuer Dringlichkeit auf und verweist auf zwei widerstreitende Grundprinzipien, die für Gegenwartsgesellschaften konstitutiv sind: persönliche Autonomie und soziale Solidarität (Wagner 1995). Gerade weil der Prozess gesellschaftlicher Modernisierung individuelle Freiheit betont, hat sich der Wohlfahrtsstaat als ein zentrales Instrument sozialen Friedens und sozialer Inklusion erwiesen, denn er bindet die sozialen Kräfte neu, die im Zuge der Modernisierung die Einzelnen zunehmend aus ihren wechselseitigen sozialen Verpflichtungen der Pflege und Unterstützung der Nächsten entbunden haben und organisierte so soziale Sicherheit großflächig um. Dass durch dieses Arrangement das Verhältnis zwischen individueller Freiheit und staatlichen Interventionen immer wieder neu ausgehandelt werden muss, gehört zu der langen Geschichte des Wohlfahrtsstaates. Daher ist vom Wohlfahrtsstaat auch als institutionalisierte Solidarität die Rede (u. a. Gelissen 2000; Prisching 2003), obgleich damit offenbleibt, um wessen Solidarität es geht und ob Solidarität als zwischenmenschliche Handlungskategorie oder makrosoziales gesellschaftstheoretisches Konzept, das auf soziale Ordnung verweist, verwendet wird.

In diesen komplexen Modernisierungsprozessen kann Solidarität gleichermaßen unabhängige und abhängige Variable sein. Der Artikel fokussiert Letzteres und nimmt den grundlegenden Konflikt in seiner aktuellen Ausprägung zum Anlass, die Frage nach den „politischen Quellen von Solidarität“ (Banting und Kymlicka 2017) und den Solidarisierungspotentialen von sozialpolitischen Regulierungen zu stellen. Können derart abstrakte, staatlich organisierte und über Beitrags- und Steuerzahlungen formalisierte Unterstützungsbeziehungen in einer Gesellschaft überhaupt soziale Solidarität hervorbringen und wenn ja, welche Mechanismen sind dafür verantwortlich? Der Aufsatz interessiert sich also nicht für Solidarität als potenzielle Voraussetzung für oder konkrete instrumentelle Praxis im Wohlfahrtsstaat, sondern für Solidarität als dessen mögliches Resultat. Damit reiht er sich in eine Tradition der Wohlfahrtstaatsforschung ein, die sich eher auf dessen gesamtgesellschaftliche Wirkungsweisen und Lernprozesse konzentriert (Lessenich 2012). Entsprechend betrachte ich Wohlfahrtsstaaten als Ideen sozialer Ordnung, die in die Gesellschaft zurückstrahlen und potenziell neue großräumige Solidarhorizonte vermitteln. Wohlfahrtsstaatliche Politik wird so zu einer wesentlichen Voraussetzung für die Entstehung von Solidarität unter Fremden.

Im Unterschied zu kommunitaristischen Denkschulen wird Solidarität aber nicht auf vermeintlich naturwüchsige Gleichheit bezogene Lebenszusammenhänge reduziert, sondern in der Tradition der klassischen Soziologie als dynamisches und elastisches Konzept gedacht (Abschn. 2). Dieses Solidaritätsverständnis kombiniert soziologische Ansätze, die der Transformativität von Solidarität Rechnung tragen (Durkheim 1992 [1893]; Kymlicka 2015; Banting und Kymlicka 2017) mit wohlfahrstaatstheoretischen Zugängen, die den Wohlfahrtsstaat als institutionalisierte Solidarität begreifen. Damit baut die Analyse auf der Annahme auf, dass Solidarität in hochgradig differenzierten Gesellschaften auf politische Steuerungsleistungen angewiesen ist. Die Formen und Erfolgsbedingungen dieser Steuerungsprozesse werden in diesen Darstellungen jedoch kaum berücksichtigt.

Im Industriekapitalismus des ausgehenden 20. Jahrhunderts geriet Sozialpolitik als eine Variante der politischen Steuerung von Solidarität zur modernen Antwort auf das Problem sozialer Ordnung. Für die Sozialintegration nationalstaatlich verfasster Marktgesellschaften stellte wohlfahrtsstaatliche Politik fortan eine wesentliche Stellschraube und Antriebskraft dar (Marshall 1992 [1949]; Lessenich 2008, S. 35 ff.). Daran anknüpfend betrachtet der Beitrag die Solidarisierungspotentiale moderner Wohlfahrtspolitik aus soziologisch-institutionalistischer Perspektive und fragt nach den vermittelnden Mechanismen zwischen den wohlfahrtstaatlichen Programmen einer politischen Ordnung und der Solidarität ihrer Mitglieder. Hierzu stellt Abschn. 3 die zentralen institutionentheoretischen Annahmen vor und arbeitet drei solidaritätsrelevante Wirkmechanismen heraus: die Kompassfunktion, die Stabilisierungsfunktion und die Scharnierfunktion. Das Theoriemodell wird anschließend anhand der Sozialgeschichte und Funktionsweise des deutschen Wohlfahrtsstaates beispielhaft angewendet, obgleich nicht in einem hypothesentestenden, sondern empirisch plausibilisierenden Sinn unter Zuhilfenahme der soziologischen und sozialhistorischen Wohlfahrtsstaatsforschung. Mit der Gesetzlichen Krankenversicherung (GKV) und dem Arbeitslosengeld 2 nach SGB II fokussiert die Untersuchung zwei einschlägige wohlfahrtsstaatliche Instrumente, so dass das gesellschaftstheoretische Argument über die beiden den deutschen Sozialstaat repräsentierenden Instrumente der Versicherung und der sozialen Grundsicherung illustriert werden kann (Abschn. 4).

Der institutionentheoretische Zugriff erlaubt es die Makro- mit der Mikroebene derart zu verbinden, dass einerseits die Orientierungen der innerhalb wohlfahrtsstaatlicher Organisationen und Sozialverwaltungen agierenden Personen deutlich werden und andererseits individuelle Wertvorstellungen und solidaritätsbezogene Einstellungen adressiert werden können. Letztere werden anhand von Sekundärdaten auf der Aggregatebene dargestellt. Das Vorgehen verspricht, widersprüchliche gesamtgesellschaftliche Zeitdiagnosen (wie Polarisierung vs. neue Solidaritäten) und ambivalente bis krisenhafte Sozialstaatsdeutungen besser einordnen und aufeinander beziehen zu können und macht den Untersuchungsgegenstand ‚Wohlfahrtsstaat‘ anschlussfähig an gesellschaftstheoretische Fragestellungen.

## Solidarität als Beziehung und Prozess: Begriffliche Grundlegung

Der vorliegende Beitrag fokussiert interpersonelle Solidarität als potenzielles Ergebnis politischer Steuerungsleistungen. Im modernen Sprachgebrauch meint Solidarität die „Idee eines wechselseitigen Zusammenhangs zwischen den Mitgliedern einer Gruppe von Menschen“ (Bayertz 1998, S. 11). Damit lassen sich zwei Merkmale für solidarische zwischenmenschliche Beziehungen näher bestimmten: Reziprozität und Normativität.

Reziprozität bezieht sich auf das Strukturmerkmal solidarischer Beziehungen, das sich als wechselseitiger „organisierter Risikoausgleich“ beschreiben lässt (Hengsbach 1999, S. 36; Fehmel 2020). In modernen Gesellschaften ist die gegenseitige Erwartungshaltung nicht an konkrete Solidaritätsgeber:innen geknüpft, sondern in Bezug auf das „anonyme Kollektiv“ verallgemeinert (Dallinger 2009, S. 160). „Latente Reziprozität“ (Bayertz 1998, S. 43) meint daher die regelmäßigen gegenseitigen Unterstützungsbeziehungen zwischen einander unbekannten Mitgliedern einer Gesellschaft. Diese „*gegenseitige* Erwartung von Hilfe im Bedarfsfall“ ist für Solidarbeziehungen bezeichnend, da die beiden beteiligten Parteien prinzipiell gleichgestellt sind (ebd., Hervorhebung im Original).

Das zweite Merkmal betrifft die normative Qualität der reziproken Beziehungen. Kaufmann (2009, S. 366) begreift die Vorrangigkeit des gesellschaftlichen gegenüber dem individuellen Nutzen als konstitutives Element von Solidarität. Das bedeutet nicht, dass eine wie auch immer geartete Solidarbeziehung nicht auch individuelle Interessen bedienen kann, verdeutlicht aber, dass sich die Solidaritätsbeziehungen innerhalb einer Gesellschaft im Spannungsfeld individueller und kollektiver Interessen aufspannen. Analytisch ist es demzufolge sinnvoll, zwischen den individuellen Motiven, einer Solidarbeziehung beizutreten oder diese zu akzeptieren (bspw. sich für zukünftige Notlage absichern zu wollen) und dem kollektiven Nutzen, der aus solchen Beziehungen erwächst, zu unterscheiden. Unterschiedliche und zum Teil sogar widersprüchliche Formen von Solidarität lassen sich so in einem Kontinuum zwischen Eigeninteressen und Gemeinschaftsinteressen verorten. In diesem Spannungsfeld individueller und kollektiver Interessen ist Solidarität makrosoziologisch häufig zum notwendigen sozialen Bindemittel und „Zement“ (Bayertz 1998, S. 11) erklärt worden. Für Durkheim stellte Solidarität ein gesamtgesellschaftliches Ordnungsprinzip dar, das den Zusammenhalt in zunehmend ausdifferenzierten, national (wie auch transnational) verfassten Gesellschaften erleichtert. Mit zunehmender Größe und Komplexität der sozialen Ordnung ist die Frage, worauf dieser wechselseitige Zusammenhang genau beruht oder wie geteilte Normen entstehen, jedoch immer schwerer zu beantworten. Mit wachsender gesellschaftlicher Heterogenität, das heißt, „wenn das Kollektivbewusstsein seine Basis in der Gleichheit verliert“, ist das Individuum verstärkt gefordert, die Unterschiede durch „nachgeschobene Bewußtseinsleistungen“ zu kompensieren und sich zu anderen ins Verhältnis zu setzten (Durkheim 1992 [1893], S. 26). Durkheims makroanalytisches Verständnis von Solidarität rückt damit in die Nähe von überindividuellen Begrifflichkeiten wie sozialer Zusammenhalt oder Kohäsion, während das Handlungsprinzip Solidarität die komplexen interpersonellen Interdependenzen moderner Gesellschaftlichkeit betont.

Der beobachtete Übergang von segmentären zu funktional organisierten Gesellschaften, mit dem sich laut Durkheim auch der vorherrschende Solidaritätsmodus ändere, legt zudem ein dynamisches Solidaritätsverständnis nahe. Es stellt die Voraussetzung für die potenzielle Möglichkeit einer Solidarität unter Fremden dar. Ähnlich wie Durkheim das empirisch am Beispiel des spezifischen Rechtstypus und der Arbeitsteiligkeit darlegte, lässt sich das auch für den historischen Wandel sozialer Sicherungssysteme zeigen. Fiel die Aufgabe der sozialen Sicherung traditionell in den gemeinschaftlichen Bereich und war kleinräumig organisiert (familial, nachbarschaftlich, berufsständisch), wurde sie am Ende des 19. Jahrhunderts zusehends zur Staatsaufgabe. Im Zuge der Herausbildung nationaler Wohlfahrtstaaten wurden die reziproken Verantwortungsübernahmen bei sozialen Notlagen über den sozialen Nahbereich hinaus ausgeweitet. Auch wenn das jeweilige Solidaritätsverständnis sozialem Wandel unterliegt, hat sich infolge dieses Prozesses seit der Nachkriegszeit ein wirkmächtiges Narrativ des Wohlfahrtsstaates als institutionalisierte Solidarität herausgebildet, demzufolge zahlreiche Autor:innen den Wohlfahrtsstaat als „moderne Verkörperung von Solidarität“ (Prisching 2003, S. 157; Gelissen 2000; Ullrich 2000) bzw. „organisierte Solidarität“ (Dallinger 2009, S. 214) betrachten.^2^ „Er konstituiert – wie in einer (über)großen Familie – einen gegenseitigen Risiko‑, Schadens- und Bedarfsausgleich unter den Mitgliedern der (gesellschaftlichen) Gemeinschaft“ (Lessenich 2008, S. 33).

Trotz der konzeptuellen Elastizität neigt das wohlfahrtsstaatliche Solidaritätsnarrativ dazu, Solidarität als monolithische Veranstaltung zu begreifen, was in zweierlei Hinsicht zu analytischen Widersprüchen führt. Zum einen vermag es die Lesart des Sozialstaates als institutionalisierte Solidarität nicht, zwischen unterschiedlichen analytischen Ebenen zu unterscheiden. Dadurch bleibt offen, ob sich Solidarität im sozialstaatlichen Kontext auf die Motive der Handelnden, auf die Instrumente selbst oder auf die abstrakten Leitideen, die sozialpolitisches Handeln anleiten, bezieht. Zum anderen tendiert das Solidaritätsnarrativ dazu, wohlfahrtsstaatliche Selbstbeschreibungen wie das Solidarprinzip für bare Münzen zu nehmen, ohne den Sinngehalt dieser vermeintlichen Solidarität zu hinterfragen. Diese auf einen sozialen Ausgleich zwischen unterschiedlichen sozialen Gruppen abhebende abstrakte Idee des Solidarprinzips muss jedoch in konkrete Handlungskategorien und Praktiken übersetzt werden.

Mit Blick auf diese beiden Schwachstellen und da eine Soziologie des Sozialstaates dem Anspruch nach über die Beobachtung der Selbstbeschreibungen hinausgeht (Kaufmann 2009), rückt der vorliegende Beitrag die wohlfahrtsstaatlichen Mechanismen und damit die Voraussetzungen solidarischen Handelns in modernen Wohlfahrtsgesellschaften in den Mittelpunkt. Mithilfe des institutionentheoretischen Zugriffs lassen sich diese Widersprüche auflösen, indem Solidarität als Leitidee, die sozialpolitisches Handeln anleitet und wiederum selbst neue Solidaritäten hervorbringen kann, gefasst wird. Damit ist neben den horizontalen und vertikalen Beziehungen einer Gesellschaft eine dritte normative Begriffsfacette von Solidarität angesprochen, auf die weiter unten zurückzukommen sein wird.

## Wie kann Sozialpolitik soziale Solidarität hervorbringen? Ein Vorschlag zur Theoretisierung

Der institutionentheoretische Ansatz, der im Folgenden vorgestellt wird, erlaubt es, die vermittelnden Mechanismen zwischen den wohlfahrtstaatlichen Programmen einer politischen Ordnung und der potenziellen Solidarität ihrer Mitglieder zu spezifizieren. Der Wohlfahrtsstaat wird dabei nicht mit Solidarität gleichgesetzt, sondern als institutionalisierte „Ordnungsidee“ (Lepsius 1990, S. 76) und kollektiv ausgehandelte Verhaltenssteuerung betrachtet, die ein Fundament für die solidarischen Beziehungen zwischen den Mitgliedern einer Gesellschaft darstellen kann. Dieser Analysefokus schließt selbstverständlich nicht aus, dass es parallel dazu auch andere Quellen gesellschaftlich relevanter Solidarität gibt.

Mittels Institutionen werden Vorstellungen von sozialer Ordnung in handlungswirksame Regelwerke, Verhaltensnormen und -muster gegossen. Kultursoziologisch geprägten institutionalistischen Theorien zufolge stellen Institutionen „Sozialregulationen“ dar, „in denen Prinzipien und Geltungsansprüche einer Ordnung symbolisch zum Ausdruck gebracht werden“ (Rehberg 2014, S. 53). Anknüpfend an diese Definition betrachtet Lepsius Institutionen als Prozesse, „die soziales Verhalten strukturieren und auf Wertvorstellungen beziehen“, sogenannte Leitideen (Lepsius 1997a, S. 58). Institutionen verkörpern also Leitideen, die in ihrem jeweiligen Geltungsbereich verhaltensrelevant sind. Als „institutionalisierte[s] ‚Gegenprinzip‘ zum Kapitalismus“ (Lepsius 2013, S. 20) bildet Sozialpolitik einen solchen ausdifferenzierten Geltungskontext, innerhalb dessen das Verhalten der Akteure in Bezug auf die Verteilung von für soziale Teilhabe wichtigen Ressourcen gesteuert wird. Damit adressieren sozialpolitische Institutionen Verteilungs- und Anerkennungsprobleme, deren Bearbeitung wiederum in andere Institutionen zurückstrahlt. Bspw. wirkt sich die im Ehegattensplitting verankerte Leitidee der Förderung der Ehe auf die innerfamiliäre Arbeitsteilung sowie Rollenbilder etc. aus und stabilisiert zugleich die Ehe als Institution.

Neben kulturellen Leitideen und sozialen Institutionen, in denen diese verankert sein können, unterscheidet Lepsius zudem Organisationen als Trägerinnen und Vermittlerinnen von institutionalisierten Leitideen. Durch Akteurshandeln in Organisationen werden Institutionen überhaupt erst zum Leben erweckt. Diese Unterscheidung erlaubt es analytisch zwischen der sozialstaatlich institutionalisierten Leitidee, konkreten sozialpolitischen Instrumenten und Maßnahmen und den organisationalen Apparaten, in denen diese umgesetzt werden, zu trennen, um institutionenanalytisch „das Spannungsfeld zwischen Ideen und Verhaltensstrukturierung zu beschreiben und zu erklären“ (Ebd., S. 14).

Trotz gesellschaftlicher Beharrungskräfte stellen soziale Institutionen keine unabänderlichen Gewissheiten dar, sondern sind Gegenstand permanenter Aushandlungsprozesse. Denn „durch den Umstand, dass ein Leitbild immer auch diffus bleibt, ist es für die Vielzahl an beteiligten Subjekten anschlussfähig. […] Die Subjekte befinden sich durch die wandelbaren Leitbilder in einem wiederkehrenden unabgeschlossenen Interpretationsprozess“ (Bohmann und Lindner 2020, S. 37). Die oben geäußerte Kritik aufgreifend lässt sich das wohlfahrtsstaatliche Solidaritätsnarrativ so genauer bestimmen. Dient es einerseits als normativer Ankerpunkt in parteipolitischen und parlamentarischen Debatten, ist die institutionalisierte Leitidee der Solidarität andererseits selbst sozialem Wandel ausgesetzt und muss sich gegen widerstreitende Leitideen durchsetzen und neu erfinden.

Um die Frage zu beantworten, welche Auswirkungen Institutionen und die ihnen impliziten normativen Leitideen haben, muss schließlich noch geklärt werden, in welchem Verhältnis Individuen und Institutionen zu einander stehen. Als steuernde Regelwerke und verbindliche „kulturelle Vermittlungsinstanzen zwischen Sozialstruktur und Sinnproduktion“ (Rehberg 1997, S. 103) leiten Institutionen individuelle Handlungen an und steuern Motive. Personen reagieren jedoch nicht einfach auf die vorgefundenen Ideen und Normen, sondern prägen diese selbst mit, so dass permanente (De‑)Institutionalisierungsprozesse entstehen. Ihre Verbindlichkeit beruht auf den jeweiligen Leitideen, „die als Deutungsrahmen für bestimmte Bereiche normative Gültigkeit erlangt haben“ und so dazu beitragen, Unsicherheit in gemeinsamen Handlungskontexten abzubauen, indem die Beteiligten auf „spezifische Wertorientierungen und Handlungsstrukturierungen“ hin zentriert werden (Lepsius 1997a, S. 60). Bohmann und Lindner (2020, S. 37) zufolge tragen Individuen dadurch nicht nur dazu bei, Institutionen aufrechtzuerhalten, sondern sie auch weiterzuentwickeln. Das Verhältnis zwischen Institutionen und Individuen-ist also kein einseitiger direktiver Prozess, sondern eine wechselseitige Beziehung, bei der bspw. individuelle Autonomievorstellungen Leitideen verändern können.

In ihrem dynamischen Zusammenspiel aus Leitideen, Institutionen und Organisationen strukturieren sozialpolitische Institutionen das Bewusstsein und Verhalten Einzelner und prägen so mittel- und langfristig deren individuelle Präferenzen und Einstellungen (bspw. in Bezug auf Umverteilungspräferenzen oder Gerechtigkeitsvorstellungen). Die These der Verhaltenswirksamkeit von Institutionen lenkt die Aufmerksamkeit auf deren Effekte. Entsprechend lautet die These dieses Artikels, dass sozialpolitische Institutionen Solidarisierungspotentiale hervorbringen können. Solidarität, so das Hauptargument, kann dadurch zur unmittelbaren Folge von Sozialpolitik werden. Die Geltungsmacht und Erlernbarkeit der institutionalisierten sozialpolitischen Leitideen ermöglichen Interdependenzerfahrungen, welche die Bereitschaft zur Solidarität begünstigen. Aber wie genau erwächst aus Sozialpolitik zwischenmenschliche Solidarität und welche Mechanismen sind für die solidaritätsstiftende Wirkung verantwortlich?

Mit den eben eingeführten institutionentheoretischen Kategorien Leitidee, Institution, Organisation korrespondieren drei Gestaltungsebenen, die als Quelle sozialer Solidarität fungieren. Die naheliegendste Quelle stellen auf der normativen Ebene die den wohlfahrtsstaatlichen Institutionen impliziten Leitideen selbst dar. Eine stark abstrahierte Quelle von Solidarität stellt zweitens die (sozial)rechtliche Steuerungsebene dar, welche die sozialen Beziehungen zwischen den Bürgerinnen und Bürgern reguliert. Und drittens kommt auf der organisationalen Ebene den im Rahmen moderner Wohlfahrtsstaaten installierten Sozialverwaltungen eine entscheidende Rolle in der Gestaltung der vertikalen Beziehung zwischen den Bürger:innen und dem Staat zu (Goodin 1988). Aus diesen drei Gestaltungsebenen – normativ, rechtlich, administrativ – lassen sich nun drei zentrale Wirkmechanismen ableiten, mithilfe derer die spezifischen sozialstaatlichen Solidarisierungspotentiale näher bestimmt werden können. Diese bezeichne ich als Kompass‑, Stabilisierungs- und Scharnierfunktion (vgl. Tab. 1).

**Table 1:** 

	Kompassfunktion	Stabilisierungsfunktion	Scharnierfunktion
*Gestaltungebene*	Normative Ebene	Steuerungsebene (instrumentell-rechtlich)	Administrativ-organisatorische Ebene
*institutionentheoretisch*	Leitidee	Institution	Organisation
*Analytisch*	Normativ/kulturell	Interpersonell	Organisational
*Solidarität als*	Norm und Wertorientierung (Umverteilungsbereitschaft)	Interdependenz/Zusammengehörigkeit (horizontale Beziehungen)	Zugehörigkeit/Mitgliedschaft (vertikale Beziehungen)
*Wohlfahrtstaat* *(idealtypisch)*	Wohlfahrtsstaatlich organisierte Solidarnormen	Pflichtversicherung u. Ä.; Rechtsanspruch auf Leistungen	Staat als Adressat von Ansprüchen
*Modernisierungstheoretische Dysfunktion*	Individualisierung, konkurrierende Leitideen	Soziale Disparitäten, Diversitätsdruck	Ausbalancieren von Rechten und Pflichten

Quelle: Eigene Darstellung

Die *Kompassfunktion* bezieht sich auf die normative Ebene sozialstaatlicher Politik und betrachtet Sozialpolitik als Normgeberin. Sie betont deren Verhaltenswirksamkeit, die sich aus der Orientierungsfunktion von Werten ergibt. Nimmt man die von Lepsius konstatierte verhaltensprägende Wirkung von Institutionen ernst, dann dienen die institutionalisierten Wertorientierungen im Idealfall als sozialisatorische Werte und Blueprints für solidarisches Verhalten. So institutionalisieren sozialpolitische Programme bspw. Verhaltensorientierungen, welche die an gewisse Voraussetzungen und Pflichten geknüpfte gegenseitige Unterstützung ihrer Adressat:innen vorsieht.

Die überlappenden sozialpolitischen Adressat:innenkreise verinnerlichen die von den sozialpolitischen Institutionen mindestens implizit mitgeführten Leitideen und Wertorientierungen, denn „ohne legitimierende Wertbindungen können Institutionen nicht dauerhaft aufrechterhalten werden“ (Lepsius 1990, S. 63). So entsteht schließlich ein normativer Rahmen, anhand dessen Akteure nicht nur ihre Erwartungen, sondern auch ihre individuellen Umverteilungspräferenzen, Deutungsmuster und Gerechtigkeitsvorstellungen ausrichten, die ihnen als normative Basis für die Beantwortung der Frage wer, wann, wie, wem gegenüber solidarisch ist, dient. Die Kompassfunktion bezieht sich damit nicht nur auf abstrakte Leitideen (etwa Bedarfssicherung oder die der Institution der Familienversicherung implizite Idee des inner- und interfamilialen Ausgleichs), sondern umfasst auch die instrumentelle Ebene, auf der die Reichweite, die Intensität und die Voraussetzungen der gegenseitigen Unterstützungsbeziehungen konkretisiert werden. Dem Wohlfahrtsstaat wird damit eine sekundäre gesamtgesellschaftliche Wirkung zuteil, die weit über dessen makro- und sozioökonomischen Effekte und stratifizierenden Wirkungen hinausgeht. Unterstützt wird dieser normative Rahmen durch die ins kulturelle Repertoire einer Gesellschaft eingehenden historischen Narrative und öffentlichen Diskurse, durch die sozialstaatliche Wertideen, Leitbilder und Deutungsmuster Verbreitung finden und so individuelle „socially embedded preferences“ (Brooks und Manza 2007, S. 7) mitprägen. Dieses Argument wird auch von der wohlfahrtsstaatlichen Einstellungsforschung gestützt, die gezeigt hat, dass sozialpolitische Programme und die ihnen impliziten Wertorientierungen individuelle Einstellungen hinsichtlich sozialstaatlicher Unterstützungsniveaus und die allgemeine Zustimmung zum Wohlfahrtsstaat prägen (Rothstein 1998). Auch die Frage, welche Personengruppen zu welchen Konditionen staatliche Unterstützung erhalten sollen, ist eng mit den historisch gewachsenen wohlfahrtsstaatlichen Leitideen verknüpft sind (Mau 2003; Streensland 2006).

Dass die historisch gewachsenen wohlfahrtsstaatlichen Institutionen über die Jahrzehnte eine deutliche normative Prägekraft entwickelt haben, ist unumstritten. Dennoch ist dieser Prozess selbstverständlich kein Automatismus und läuft nicht einheitlich ab. Denn modernisierungstheoretisch betrachtet können Prozesse wie Pluralisierung und Individualisierung, die eine stärkere Ausdifferenzierung individueller Einstellungen und Wertorientierungen vorangetrieben haben, zu Dysfunktionen auf der normativen Ebene führen. Dadurch hat sich die produktive Wechselbeziehung zwischen Individuum und Institution zugunsten des Individuums verschoben, das angetrieben von institutionellen Anforderungen selbst (Eigenverantwortung, Flexibilität usw.) zunehmend persönliche Freiheitsansprüche an die Institutionen heranträgt (Börner et al. 2020). Folglich spricht Vieles dafür, dass die Leitideen einerseits vor dem Hintergrund sozioökonomischer Faktoren und persönlicher Präferenzen (um)interpretiert werden und andererseits auch konkurrierende Leitideen Eingang in das individuelle Wertsystem finden und priorisiert werden können.

Die *Stabilisierungsfunktion* umfasst die Steuerungsebene und fragt, auf welche Art und Weise soziale Rechte und sozialpolitische Instrumente die horizontalen Beziehungen zwischen den Mitgliedern einer Gesellschaft strukturieren sowie nach dem Vertrauen in diese formalisierten Beziehungen. So macht es einen Unterschied, ob sozialpolitische Maßnahmen über die freiwillige Selbstverpflichtung der Beteiligten gesteuert werden oder als Rechtsanspruch zur Verfügung stehen. Sozialpolitische Maßnahmen unterscheiden sich auch hinsichtlich der Durchsetzungs- und Sanktionsmöglichkeiten, etwa mittels verpflichtender Eigenleistungen wie im Fall von Sozialversicherungsbeiträgen. Die Anerkennung sozialer Rechte und die Kontroll- und Durchsetzungskraft sozialer Programme schaffen individuelle Erwartungssicherheit, nicht nur in Bezug auf die rechtliche Garantie bestimmter sozialstaatlicher Leistungen, sondern auch in Bezug auf die Tatsache, dass alle anderen Mitglieder ebenfalls regelkonform handeln (bspw. ihre Beiträge zahlen) und damit zur allgemeinen Operabilität des Gefüges beitragen. Diese „Sanktionsmacht“ (Lepsius 1997a, S. 60) von Institutionen hilft, den Geltungsanspruch einer Leitidee durchzusetzen. Soziale Sicherheit wird dadurch zu einem quasikollektiven Gut, das auf der „institutionell abgesicherte[n] Erwartung eines kontinuierlichen Einkommens in ausreichender Höhe, das entweder aus dem Arbeitsmarkt oder aus dem System sozialer Sicherung bezogen wird“, beruht (Vobruba 2009, S. 95). Auch hier spricht aus modernisierungstheoretischer Perspektive jedoch einiges dafür, dass die gewachsene Heterogenität spätmoderner Gesellschaften die Vertrauensbeziehungen zwischen unterschiedlichen sozialen Gruppen erschwert hat, wie insbesondere Studien zum Wohlfahrtschauvinismus gezeigt haben (Breznau und Eger 2016). Die wichtigste, der Stabilisierungsfunktion gegenläufige Dysfunktion liegt demzufolge in dem „Diversitätsstress“ (Mau et al. 2020), dem spätmoderne Gesellschaften ausgesetzt sind, begründet.

Die *Scharnierfunktion* schließlich bezieht sich auf die administrativ-organisatorische Gestaltungsebene von Sozialpolitik, über die sich die vertikalen Beziehungen der Bürger:innen zum Staat aufspannen, einschließlich deren symbolischer Repräsentationen. Die Vielzahl an kommunalen Behörden, Sozialverwaltungen und quasistaatlichen Organisationen machen den Wohlfahrtsstaat überhaupt erst sichtbar und erfahrbar (Kaufmann 1997a, S. 22 f.). Der wohlfahrtsstaatliche Komplex bildet ein wesentliches Element politischer Gemeinwesen, da er wie kaum ein anderer Politikbereich unmittelbare Auswirkungen auf die Lebensbedingungen der Mitglieder dieses Gemeinwesens hat und in deren Lebensgestaltung eingreift (Lessenich 2012, S. 33 f.). Dessen Trägerorganisationen und Sozialverwaltungen (wie Gesundheitsamt oder Jobcenter) bilden dann einen positiven Bezugspunkt für die Bevölkerung, wenn sie zuverlässige und transparente „Zurechnungseinheiten für Erwartungen und Ansprüche“ darstellen (Lepsius 1999, S. 213). Sie stellen Begegnungsorte mit dem Staat, an die die Bürger:innen ihre Ansprüche adressieren bzw. von denen sie sich beraten lassen, dar. Aufgrund der bewusstseinsprägenden Wirkung von Institutionen, über die potenziell sogar politische Zugehörigkeiten angesprochen werden (Lepsius 1997b, S. 949), können diese Begegnungen das Bewusstsein in Bezug auf die politische Zugehörigkeit der Bürger:innen maßgeblich mitprägen. Über den hier als Scharnierfunktion bezeichneten Mechanismus können sich also gesellschaftliche Solidaritätshorizonte formieren und in einem größeren Bezugsrahmen institutionalisieren. Im Vergleich zu den ersten beiden Solidaritätsformen vermittelt diese Funktion über die vertikalen Beziehungen die abstrakteste Variante von Solidarität, die sich auf politische Zugehörigkeit und kollektive Identität bezieht.

In dem Vermittlungsprozess spielen die konkrete Ausgestaltung des jeweiligen Instruments und die Ansprache der Bürger:innen eine Rolle. Bspw. leidet die Transparenz und Erwartungssicherheit bei sogenannten Kann-Leistungen, welche den Sachbearbeitenden besonders große Ermessensspielräume einräumen, die nicht mehr im Sinne der Einzelfallgerechtigkeit sind, sondern eher systemfremde Leitideen wie Wirtschaftlichkeit enthalten (Fehmel 2017). Zudem können die für die Stabilisierung der horizontalen Beziehungen zuträglichen Kontroll- und Sanktionsmechanismen hier eher schädlich sein.

Auch wenn repräsentative Umfragen darauf hindeuten, dass das Vertrauen in den Wohlfahrtsstaat auf einem hohen Niveau stabil ist (Roller 2002), ist der Grat zwischen dem gelungenen Maß an Rechten und Pflichten, aus vertrauensstiftenden und freiheitsbeschränkenden Maßnahmen schmal. Das hat Habermas (1981, S. 530 ff., 1998, S. 133) dazu veranlasst, eine gesellschaftstheoretisch hergeleitete Sozialstaatskritik zu formulieren, die die „normalisierende Gewalt von Sozialbürokratien“ aufgrund ihrer massiven lebensweltlichen Interventionen hervorhebt. Demgegenüber betrachten sozialhistorische Lesarten des Wohlfahrtsstaates diese Einschnitte nicht als absolute Autonomieverluste, da sie „lediglich die Ebene der sozialen Kontrolle“ und Abhängigkeit aus dem sozialen Nahbereich herauslösen und auf Sozialbürokratien verlagerten (Alber 1982, S. 62; Goodin 1988, S. 174). Ähnlich argumentiert auch Marshall (1992 [1949]), wenn er die autonomiestiftende Wirkung von Sozialpolitik betont. Dysfunktional für den Vermittlungsprozess wird dieser Deutungskonflikt um das Verhältnis von wohlfahrtsstaatlichen Interventionen und individueller Autonomie immer dann, wenn aus Sicht der Adressat:innen von Sozialpolitik das aufgebrachte Maß an Kontrolle oder Sanktionen nicht mehr im Verhältnis zu den Rechten bzw. staatlichen Leistungen steht und folglich als Verschiebung in Richtung Unrecht gedeutet werden.

Mit diesem theoretischen Instrumentarium lässt sich nun die These des Aufsatzes spezifizieren, dass sozialpolitische Institutionen gesellschaftsrelevante Wirkungen nicht nur in politischer und wirtschaftlicher Hinsicht zeitigen, sondern auch im Hinblick auf die Unterstützungsbereitschaft und soziale Solidarität der Gesellschaftsmitglieder untereinander und in Bezug auf das Staatswesen. Diese Auswirkungen entfalten sich langfristig über die drei erläuterten Mechanismen, die in Abhängigkeit voneinander die normativen Orientierungen und vertikalen wie horizontalen Beziehungen prägen. Damit ist keineswegs gesagt, dass sozialpolitischen Institutionen Prinzipien wie Wirtschaftlichkeit und Effizienz völlig fremd sind. Sie sind aber nicht die einzigen und in vielen Fällen auch nicht die handlungsleitenden wohlfahrtsstaatlichen Ideen. Daher wäre es auch theoretisch unbefriedigend zu unterstellen, dass jede sozialstaatliche Institution automatisch positive Erfahrungen oder Anknüpfungspunkte hervorruft, die sich in Solidarität übersetzen. Entsprechend beleuchtet die folgende beispielhafte Institutionenanalyse auch die potenziell dysfunktionalen oder destruktiven Tendenzen am Beispiel des deutschen Sozialstaates, welche etwa durch intransparentes Organisationenhandeln oder konfligierende Leitideen, die systemisch und individuell nicht mehr bearbeitet werden können, hervorgerufen werden.

## Solidaritätseffekte im deutschen Wohlfahrtsstaat

Institutionentheoretisch stellt der Wohlfahrtsstaat eine „Ordnungsidee“ (Lepsius 1990, S. 76) dar, die kulturelle Wertvorstellungen institutionalisiert, soziale Konflikte einhegt und Interdependenzgefüge zwischen den Adressat:innen wohlfahrstaatlicher Programme etabliert. Im Folgenden wird daher der Versuch unternommen, beispielhaft die Konstitutionsbedingungen bundesdeutscher Sozialstaatlichkeit und die Funktionsweise dieser Ordnungsidee institutionenanalytisch anhand der drei Mechanismen zu skizzieren.

Die *Kompassfunktion* verweist auf den wohlfahrtsstaatlich abgesteckten normativen Rahmen, der unter anderem für die Frage der Umverteilungsbereitschaft, sprich der Reichweite von Solidarität, entscheidend ist. Dies lässt sich an der Entwicklung des gesetzlichen Krankenversicherungswesens in Deutschland illustrieren, das die großflächige Institutionalisierung des Versicherungsgedankens beispielhaft widerspiegelt. Die 1889 unter Bismarck verabschiedete Pflichtversicherung war zunächst nur für die Gruppe der Arbeitenden vorgesehen. Bismarck übernahm das bestehende Kassenwesen und griff damit zunächst die Homogenität betonende berufsgenossenschaftliche Organisation auf (Tennstedt 1983). Zwar wurde der Kreis der Versicherten bald schon weiter ausgedehnt. Aufgrund der Differenzierung schien es jedoch Anfang des 20. Jahrhunderts noch undenkbar, dass Angestellte und Arbeiterschaft in eine Kasse einzahlten (Vgl. Kott 2014). Diese Fragmentierung charakterisiert bis heute das Sozialversicherungswesen in Deutschland, wenn auch in deutlich abgeschwächter Form. Im deutschen Kontext ist das Solidaritätsverständnis von der historisch gewachsenen Spaltung des Krankenversicherungssystems in gesetzliche und private Kassen, die implizit die Leitidee einer nach Leistungsklassen fragmentierten sozialen Gerechtigkeit mit sich führen, geprägt. Dadurch unterscheidet es sich maßgeblich von universalen Beveridge-Modellen wie dem britischen Modell. Es sind insbesondere zwei auch gesellschaftspolitisch relevante Sollbruchstellen im Verantwortungsgefüge der GKV, die in den deutschen Reformdebatten immer wieder zu Tage treten: Das ist zum einen die Exit-Option der Privaten Krankenversicherung für Besserverdienende und zum anderen die beamtenrechtliche Beihilfe. Entsprechend werden sie häufig als Solidaritätsbrüche wahrgenommen – ein Begriff, der auf einen unerfüllten Solidaritätsanspruch seitens der staatlich versicherten Gruppen hindeutet.

Neben der Frage der Reichweite der Solidarität erfüllt der deutsche Sozialstaat seine Kompassfunktion insbesondere über verschiedene institutionalisierte Leitideen wie Solidarität oder Eigenverantwortung und Bedarfs- oder Leistungsgerechtigkeit, die als verhaltenswirksame Wertorientierungen fungieren. Sie dokumentieren historisch gewachsene und kulturell tief verankerte gesellschaftliche Wert- und Gerechtigkeitsvorstellungen.

Eine der Leitideen der GKV ist das Bedarfsprinzip, das im Unterschied zum lebensstandardsichernden Äquivalenzprinzip der Rentenversicherung nicht die individuelle Leistungsfähigkeit, sondern den Bedarf an medizinischer Versorgung und therapeutischen Mitteln in den Vordergrund stellt. Dadurch sind die Anforderungen an die Solidaritätsbereitschaft der Versicherten auch in Pflichtversicherungssystemen vergleichsweise hoch (Ullrich 2000, S. 11). Abstrakte Leitideen werden über die konkrete Ausgestaltung der einzelnen Instrumente, welche die Selektivität, Konditionalität und Höhe der Leistungen definieren, spezifiziert. Am Beispiel des Bedarfsprinzips wird deutlich, dass es hierbei auch zu Überschneidungen mit anderen Wertvorstellungen kommt. So zeigt sich bspw. in der Institution der Familienversicherung nach SGB V, § 10 nicht nur der solidarische Gedanke eines bedarfsgerechten Poolens von Risiken, das auch Personen einbezieht, die selbst keine finanziellen Beiträge zur GKV leisten, sondern auch der Stellenwert der Familie (deren Definition selbst sich über die Jahrzehnte verschiebt) als zentraler Reproduktionseinheit der Gesellschaft.

Insgesamt lag die Zustimmung zu sozialstaatlicher Umverteilung in Deutschland 2016 zwischen 83 % (bei Arbeitslosigkeit) bis 97 % (Krankheit) (Gerhards et al. 2018, S. 19 ff.), was für ein sehr hohes Maß an (zumindest abstrakter) institutionell vermittelter Solidaritätsbereitschaft innerhalb der Bevölkerung spricht. Die starke Korrespondenz kultureller Leitideen und persönlicher Umverteilungspräferenzen bröckelt jedoch, wenn man die individuellen wertbezogenen Einstellungen differenzierter betrachtet. So variiert die Solidaritätsbereitschaft innerhalb der deutschen Bevölkerung einerseits stark zwischen unterschiedlichen Sicherungssystemen entlang sogenannter Deservingnesskriterien.^3^ Andererseits wird der Einfluss der sozialstaatlich institutionalisierten Leitideen auf die Solidaritätsbereitschaft durch einen sozialen Gradienten auf der Mikroebene abgeschwächt (Lux et al. 2022). Zudem erschweren konkurrierende, eher neoliberal ausgerichtete Leitideen wie die der Eigenverantwortung, die zuletzt stark an Einfluss gewonnen haben, die Ausbildung von solidarischen Einstellungen (etwa Fraser 2017).

Bei der *Scharnierfunktion* geht es um die Frage, an wen die Bürgerinnen und Bürger ihre Versorgungsansprüche und politischen Forderungen adressieren können und wie verlässlich und transparent die ausführenden Organisationen sind. Entsprechend kommt hier den wohlfahrtstaatlichen Sozialverwaltungen wie Krankenkassen, Arbeitsämtern oder Arbeitsschutzbehörden eine hohe Bedeutung zu, weil sie Schnittstellen zwischen den Bürger*innen und dem Staat bzw. der Sozialgesetzgebung darstellen.

Die wohlfahrtsstaatliche Verwaltungspraxis hat zahlreiche neue Sozialpraktiken und politische Handlungskategorien hervorgebracht, die das politische Bewusstsein der sozialpolitischen Adressat*innen prägen (Wagner und Zimmermann 2003, S. 250). Historisch hat der Wohlfahrtsstaat das Armenwesen abgelöst und einen neuen Modus der Existenzsicherung etabliert. War das Armenwesen „als Gegensatz zur Staatsbürgerschaft konzipiert“ (Hockerts 1996, S. 29), werden im Wohlfahrtsstaat soziale Rechte zu einer wichtigen Säule im staatsbürgerlichen Rechtekatalog (Marshall 1992 [1949], S. 49 f.).

Der Rechtsanspruch auf soziale Sicherheit wurde in der BRD durch den sozial- und rechtsstaatlichen Verfassungsgrundsatz und die 1954 eingeführte Sozialgerichtsbarkeit, im Rahmen derer ein Rechtsanspruch auf existenzsichernde Leistungen formuliert wurde, institutionalisiert. So ist die „zwischen Staat und Gesellschaft vermittelnde Sozialpolitik zu einem prägenden Merkmal der deutschen Gesellschaftsentwicklung geworden“ (Kaufmann 2003, S. 258).

Sozialhistorische Studien zeigen, dass die Begegnung mit dem Staat selbstermächtigende Strukturen und solidarische Praktiken hervorgebracht haben. So bilden auf regionaler Ebene die von den Versicherten selbstverwalteten Krankenkassen Schnittstellen zwischen dem Staat und seinen Bürger:innen. Bereits nach der Einführung der GKV 1883 stellten die Gewerbeaufsicht, die Selbstverwaltungstätigkeiten oder die Anwendung aber auch Auslegung von gesetzlichen Regelungen Praktiken dar, die eine Selbstermächtigung der Arbeitenden und Aneignung des Staates ‚von unten‘ ermöglichten (Kott 2014, S. 16), indem sie die Anrufung staatlicher Vertreter:innen und Organe zu einer alltäglichen Praxis machten.

Die Etablierung eines Staat-Bürger:innen-Verhältnisses – und damit die Herausbildung von Staatsbürger:innen – hat nicht nur die Mobilisierung der Arbeiterinteressen beflügelt, sondern auch zur Herausbildung nationaler Identitäten mit beigetragen, wie die historische Wohlfahrtsstaatforschung gezeigt hat. Denn bei der Errichtung einer nationalstaatlichen sozialen Ordnung spielte der Wohlfahrstaat eine wichtige Rolle „by reshaping national identities, solidarities and the notion of nation state citizenhsip“ (Kettunen 2022, S. 2; vgl. auch Wagner und Zimmermann 2003; Ferrera 2005). Die so entstandenen vertikalen Beziehungen markieren politische Zugehörigkeit und konstituieren dadurch eine Form der Solidarität, die sich stark über kollektive Identitäten und Zugehörigkeit definiert. Die Scharnierfunktion hilft entsprechend, das kollektive (Selbst‑)Verständnis darüber, wer soziale Sicherheit gewährleistet, näher zu bestimmen: Also einerseits, welcher *Wohlfahrtsproduzent* (also ob soziale Sicherheit überhaupt ein öffentliches Gut ist oder durch private oder privatwirtschaftliche Akteure organisiert wird) und andererseits welche *politische Ebene* zuständig ist.

Eine empirische Annäherung an diesen vertikal vermittelnden Wirkmechanismus stellen die Akzeptanz des Sozialstaates und das Vertrauen in dessen Einrichtungen dar. Die Akzeptanz des deutschen Sozialstaates galt lange Zeit als Konsens und lag repräsentativen Bevölkerungsumfragen zufolge zwischen 1976 (bzw. 1990) und 2000 bei durchschnittlich 89 % in den alten und 96 % in den neuen Bundesländern.^4^ Im Befragungszeitraum erodierten die Werte nur leicht und lagen im Jahr 2000 schließlich bei 85 bzw. 93 %. Eine Studie aus dem Jahr 2004 lässt zudem differenziertere Aussagen über das Vertrauen in einzelne Institutionen zu. Der GKV vertrauten 2004 42,6 % der Befragten, der damaligen Sozialhilfe knapp 40 %, der Arbeitslosenversicherung ein Drittel und der Rentenversicherung nur knapp 25 % (Ullrich 2008, S. 102). Entsprechend wünschten sich die Befragten, dass der Sozialstaat Einkommensunterschiede besser ausgleiche, Familien stärker unterstütze und Arbeitsplätze garantiere. Ullrich schlussfolgert: „Nicht die Akzeptanz des bestehenden wohlfahrtsstaatlichen Arrangements ist hoch, sondern die der Wohlfahrtsstaatlichkeit“ (Ebd., S. 124). D. h. nicht die historisch gewachsene sozialpolitische Verantwortung des Staates steht in Frage, sondern die gültigen Institutionen.

Während diese Akzeptanzprobleme von einer gesteigerten Problemwahrnehmung der Bevölkerung hinsichtlich der seit den 1980er-Jahren unter ständigem Reformdruck stehenden sozialpolitischen Institutionen zeugen, deuten sie aus Sicht der Leistungsbeziehenden darauf hin, dass Begegnungen mit Verwaltungsapparaten und Street-level-Bürokrat:innen keineswegs immer positiv verlaufen bzw. autonomisierend wirken. Häufig stehen Verwaltungspraktiken und bürokratische Strukturen im öffentlichen Diskurs als intransparent oder starr in der Kritik. In der jüngeren Geschichte des deutschen Wohlfahrtsstaates haben hier besonders die arbeitsmarktpolitischen und Verwaltungsreformen im Rahmen der Agenda 2010 für Unmut gesorgt. So habe die Zusammenlegung der einstigen Arbeitslosen- und der Sozialhilfe zu einem Instrument für langzeitarbeitslose Arbeitssuchende aktivierungspolitische Widersprüche hervorgebracht:

„Die sozialverwalterischen Institutionen greifen steuernd in Lebensverläufe und Alltagspraktiken der Leute ein, legen den HilfeempfängerInnen aber individualisierte Verantwortung nahe. […] Hier wird Autonomie adressiert, der Kontrakt aber gleichzeitig als Disziplinierungsinstrument verwendet, in dem er Aufenthalt und Bemühungspflichten reguliert sowie Sanktionierungsvoraussetzungen schafft“ (Globisch 2012, S. 141).

Vergleichbare Machtasymmetrien nehmen auch Migrant:innen im Kontakt zu Sozialverwaltungen wahr. Ratzmann und Heindlmaier (2022) zufolge nehmen freiwillige Tandempartner:innen und Beratungsangebote von gemeinnützigen Organisationen in der Überweindung von Macht- und Informationsasymmetrien eine wichtige Mediationsrolle ein. Idealtypisch ist hier eine barrierefreie und diversitätssensible Ansprache der Zielgruppen sowie eine inklusive, Stigmatisierungen vermeidende Abwicklung, gefragt. Die über diese vertikalen Beziehungen zum Ausdruck kommende politische Mitgliedschaft erleichtert potenziell die Identifikation als Staatsbürger:in einschließlich der damit einhergehenden Rechte und Pflichten.

Mit der Kompass- und der Scharnierfunktion sind zwei grundlegende Mechanismen von Solidaritätsbeziehungen, angesprochen, nämlich die Einigung auf handlungsleitende solidarische Leitnormen und die Frage der Verantwortungsübernahme und Zugehörigkeit. Die konkrete Umsetzung der Leitideen und Ausgestaltung der jeweiligen sozialpolitischen Instrumente fallen in den Gegenstandsbereich der *Stabilisierungsfunktion*. Da dieser Mechanismus die horizontalen Beziehungen der Mitglieder einer Gesellschaft potenziell zu stabilisieren vermag, tritt Solidarität hier insbesondere als Interdependenzerfahrung in Erscheinung bzw. Entsolidarisierung überall da, wo sozialpolitische Regelungen Interdependenzen unterbrechen.

Die sozialpolitischen Großprojekte des „goldenen Zeitalters des Wohlfahrtsstaates“ (Flora 1986, S. XXII) brachten neuen Klassifizierungssysteme, Statuskategorien und Versorgungsklassen hervor (Lepsius 1990, S. 128 ff.), mithilfe derer sich quer durch die Gesellschaft neuartige Reziprozitäts- und Abhängigkeitsbeziehungen aufgespannt haben. Statt direkte horizontale Interaktionen ermöglichen die vom modernen Staatswesen und dessen intermediären Organisationen gestalteten bürgerlichen Beziehungen über die Institutionen des Rechts, der Bürokratie und der Demokratie indirekte und formalisierte Interaktionsbeziehungen (Ferrera 2018). Die so entstehende Solidarität zielt auf Sozialintegration ab und kommt dem gesellschaftstheoretischen Verständnis von Solidarität als abstraktem sozialem Zusammenhalt in ausdifferenzierten Gesellschaften am nächsten.

Im Laufe dieses Prozesses organisierte der sich herausbildende Wohlfahrtsstaat die zwischenmenschlichen Beziehungen großflächig neu und erweiterte damit Bezugsrahmen und Reichweite der traditionellen Sicherungsformen. Da der *face-to-face*-Kontakt der vormaligen Einheiten (insbesondere Familien, Kirchgemeinden und Vereine auf Gegenseitigkeit wie die sogenannten Arbeiterhilfskassen des Deutschen Reiches) den Sozialschutz nicht länger gewährleistete,^5^ mussten „Hilfsansprüche verrechtlicht und institutionalisiert“ (Prisching 2003, S. 169) und Erwartungshaltungen mutualisiert werden. Dadurch wurden ganz im Sinne der Stabilisierungsfunktion einander fremde Mitglieder der Gesellschaft aufeinander verpflichtet, so dass Vertrauen und Kooperation auch in einem erweiterten Umverteilungsrahmen möglich wurde (vgl. Wolf 1997). Entsprechend gelang es dem nationalen Wohlfahrtsstaat, seinen Mitgliedern „Umverteilungsopfer“ in bisher unbekanntem Ausmaß abzuverlangen (Offe 2003, S. 270). Staatliche Sozialpolitik trug so dazu bei, Solidarität trotz zunehmender Unterschiede und räumlicher Distanz auf gesamtgesellschaftlicher Ebene individuell erfahrbar zu machen, ohne dass Formen familiär oder zivilgesellschaftlicher Solidarität verdrängt würden. Gewissermaßen ergänzen wohlfahrtsstaatliche Programme damit die direkten Interaktions- durch abstrakte Interdependenzerfahrungen im nationalstaatlichen (und zunehmend auch im transnationalen) Rahmen.

Im deutschen Sozialstaat beruhte die Einübung solcher zwischenmenschlicher Solidarpraktiken stärker als in anderen Ländern auf dem „Prinzip der Sozialversicherung“ (Kaufmann 2003, S. 281). Der „doppelte Inklusionsprozess“ (Alber 1992, S. 25) der Ausweitung des Versichertenkreises und der Verbesserung des Leistungsspektrums, für die die Rentenreform von 1957 zum Symbol geworden ist, zeugt von der sozialen und stratifikatorischen Wirkmächtigkeit der sozialpolitischen Institutionen. Trotz anhaltender Klassendisparitäten und Geschlechterunterschiede prägte die Sozialpolitik dieser Jahre das öffentliche Bewusstsein und die gesellschaftlichen Beziehungen, wie sie mit zeitdiagnostischen Begriffen wie „nivellierte Mittelstandsgesellschaft“ (Schelsky 1955) und „Fahrstuhleffekt“ (Beck 1986) ausschnittweise erfasst wurden. Zugleich hat die Institutionalisierungsform der Sozialversicherung eine lohnarbeitszentrierte, männliche Arbeitsgesellschaft hervorgebracht, welche den Bezug von Lohnersatzleistungen stärker als in anderen Wohlfahrtsstaatsmodellen an zuvor geleistete Erwerbsarbeit und Beitragszahlungen koppelt (Vobruba 1990). Insbesondere die Gesetzliche Rentenversicherung sollte so „den lebenslang Erwerbstätigen die Aufrechterhaltung ihres Lebensstandards und eine Beteiligung an der fortgesetzten Produktivitätssteigerung der Wirtschaft“ sichern (Kaufmann 2003, S. 283, Hervorhebung entfernt). Im Fall derart formalisierter und verpflichtender Solidarbeziehungen äußert sich Solidarität durch die Einzelnen „im Verzicht auf das ökonomisch rationale Ausnützen illegaler Gelegenheiten oder den zweckwidrigen Gebrauch von Rechtsnormen, wie sie sich im wohlfahrtsstaatlichen Kontext vor allem als Steuerhinterziehung, Steuerumgehung und Steuerflucht, Subventionsbetrug, Schwarzarbeit oder Erschleichung von Sozialleistungen äußern“ (Kaufmann 1997a, S. 154).

Vor diesem Hintergrund können die jüngeren sozialstaatlichen Entwicklungen aus institutionentheoretischer Perspektive als De- und Re-Institutionalisierungsprozesse bezeichnet werden. Der spätestens mit dem Ende der Ära Kohl einsetzende Umbau des deutschen Sozialstaates hat unter der Maßgabe der Aktivierung und der Kosteneinsparungen bis dahin institutionenfremde Ideen wie arbeitsrechtliche Flexibilität, Deregulierung des Arbeitsmarktes und individuelle Eigenverantwortung institutionalisiert. Die zunehmende Bedeutung atypischer Beschäftigungsverhältnisse, die Abwertung lebensstandardsichernder Versicherungsleistungen bei gestiegenem Stellenwert privater Vorsorgeleistungen sowie der Einsatz von restriktiveren Sanktions- und Kontrollmaßnahmen im Leistungsrecht sind nur einige Beispiele hierfür (Butterwegge 2001; Dahme und Wohlfahrt 2003; Lessenich 2008). Auch wenn dadurch das Grundprinzip der Sozialstaatlichkeit nicht abgeschafft worden ist, haben die Reformen die sozialen Rechte und damit auch die horizontalen sozialen Beziehungen großflächig neu organisiert und wurden entsprechend vielfach als Entsolidarisierung gedeutet (Butterwegge 2001, S. 139).

De- und Re-Institutionalisierungsprozesse im Feld der Arbeitsmarktpolitik haben nicht nur aufgrund des Leistungsabbaus, sondern insbesondere aufgrund der eingeführten Sanktions- und Kontrollmaßnahmen im Rahmen der Grundsicherung für Langzeitarbeitslose das Verhältnis zwischen den Bürger:innen und dem Staat sowie zwischen den Bürger:innen untereinander neu bestimmt (Bothfeld und Betzelt 2011). Die arbeitsmarktpolitischen Maßnahmen kamen einer „Individualisierung erwerbsbezogener Chancen und Risiken“ gleich und erhöhten den Druck auf die Arbeitsuchenden, das Wohlwollen der steuerfinanzierten Solidargemeinschaft nicht zu strapazieren (Lessenich 2008). Dass die politischen Reformprozesse eine massenmediale Diskursverschiebung und öffentlichen Deutungswandel herbeigeführt haben, zeigen Metaphern und Sozialfiguren wie die der „sozialen Hängematte“, des „Sozialschmarotzers“ (Butterwegge 2014, S. 92 ff.) oder auch des „Sozialtourismus“. Der verschärfte Ton in der öffentlichen Sozialstaatsdebatte zeugt von einem gewachsenen Misstrauen zwischen den diversen sozialstaatlichen Adressatenkreisen, die sich in solchen Darstellungen als Leistungs- und Versorgungsklassen gegenüberstehen. Die überspitzten Zuschreibungen stigmatisieren komplette, teils besonders vulnerable soziale Gruppen von Leistungsbeziehenden als arbeitsscheu und betrügerisch. Mit Kaufmann, der darin eine Abnahme der sozialintegrativen Wirkung des Sozialstaates sieht, kann somit von Entsolidarisierung auf gesamtgesellschaftlicher Ebene gesprochen werden (Kaufmann 1997b).

## Fazit

Dieser Beitrag näherte sich der Frage nach der potenziellen Schlüsselrolle wohlfahrtsstaatlicher Politik für die Herausbildung belastbarer Solidarbeziehungen in Gegenwartsgesellschaften institutionentheoretisch. Hierzu wurden drei Wirkmechanismen sozialpolitischer Arrangements herausgearbeitet und am Beispiel des deutschen Sozialstaates illustriert. Auf der normativen Ebene adressieren sozialpolitische Institutionen über die in ihnen verankerten Leitideen gesellschaftliche Solidarität als Wert- und Verhaltensorientierung (*Kompassfunktion*). Vermittelt über die Steuerungsebene leistet die *Stabilisierungsfunktion* einen Beitrag zur Sozialintegration, indem sie Solidarität als horizontale soziale Beziehungen ausprägt. Über die intermediäre Rolle der Sozialadministrationen und wohlfahrtsstaatlichen Organisationen kann die *Scharnierfunktion* die Ausprägung eines kollektiven Bewusstseins und die Identifikation mit der politischen Ordnung unterstützen. Damit stellt der Wohlfahrtsstaat – ebenso wie bspw. die Zivilgesellschaft mit ihren vielfältigen Organisationsformen und Praktiken – einen wesentlichen Produktions- und Reproduktionsfaktor von Werten, Handlungspraktiken und horizontalen wie vertikalen gesellschaftlichen Beziehungen dar und macht zwischenmenschliche Solidarität zu einem Nebeneffekt wohlfahrtsstaatlicher Politik. Vermittelt über den ausgebauten Wohlfahrtsstaat des Nachkriegseuropas spannte sich in der Bevölkerung so eine dreifache Solidaritätsbeziehung auf, die hier idealtypisch dargestellt wurde. Die Beziehungen umfassen die normative Ebene (*Solidarität als Wertorientierung*) sowie die horizontalen (*Solidarität als Zusammengehörigkeit*) und vertikalen gesellschaftlichen Beziehungen (*Solidarität als Zugehörigkeit*) und betreffen dadurch je unterschiedliche begriffliche Facetten von Solidarität, die mithilfe der drei Funktionen freigelegt werden konnten (vgl. Tab. 1). Gemeinsam erfüllen die drei Wirkmechanismen so eine wichtige gesamtgesellschaftliche Integrationsfunktion. Für die Wohlfahrtstaatsforschung, die Politische Theorie und die Politische Soziologie bieten sich hier zentrale Anknüpfungspunkte, die Voraussetzungen und Quellen von Solidarität in spätmodernen Gesellschaften unter Bedingungen der Vielfalt zu denken.

Die Analyse hat jedoch auch deutlich gemacht, dass diese auf unterschiedlichen Gestaltungsebenen angesiedelten aber aufeinander bezogenen Mechanismen kein Automatismus sind, sondern nur unter bestimmten Voraussetzungen gelingen und permanenten Veränderungsdynamiken ausgesetzt sind. Greifen wohlfahrtsstaatliche Normen und Leitideen, die konkrete Ausgestaltung der sozialpolitischen Instrumente und deren organisationale Umsetzung (etwa die Ansprache wohlfahrtsstaatlicher Adressat:innen) nicht ineinander, kann der Wohlfahrtsstaat von einer Quelle gesamtgesellschaftlicher Solidarität auch zu einem Störfaktor für wechselseitige Solidarbeziehungen werden kann, wie an einzelnen Beispielen illustriert wurde.

Vor dem Hintergrund des dynamischen Institutionenverständnisses und dem für soziale Institutionen typischen komplexen „Spannungsfeld zwischen Ideen und Verhaltensstrukturierung“ (Lepsius 2013, S. 14) muss also davon ausgegangen werden, dass auch die dank der drei sozialpolitischen Wirkmechanismen etablierten solidarischen Zusammenhänge in permanenter Bewegung sind, weil sie eben nicht auf naturwüchsigen Gefühlen der Zusammengehörigkeit beruhen. Institutionentheoretisch sind zwei zentrale Erkenntnisse zu nennen, die auch zum Verständnis aktueller Widersprüche und gegenläufiger Tendenzen beitragen.

Erstens sind Institutionalisierungsprozesse andauernde Prozess, in deren Verlauf sich die Leitideen und damit auch die gesellschaftlichen Solidaritätspotentiale und Wertorientierung permanent verändern. In fortwährenden De- und Re-Institutionalisierungsprozessen tragen sozialpolitische Institutionen so die für spätmoderne Gesellschaften typischen Konflikte aus, die hier als Dysfunktionen skizziert wurden. Paradigmenwechsel wie die aktivierungspolitischen Reformen im Rahmen der Agenda 2010 sind somit besonders weitreichende Brüche mit den vorherrschenden Institutionen. Zudem erhöht das Nebeneinander unterschiedlicher und zum Teil widersprüchlicher Leitideen die Diffusität dieses Prozesses. So mögen Leitideen wie Reziprozität, Solidarität oder Bedarfsgerechtigkeit im sozialpolitischen Institutionenkomplex dominieren, sie laufen aber Gefahr aufgrund von Ökonomisierungsprozessen, im Rahmen derer widerstreitende Leitideen favorisiert werden, relativiert zu werden.

Zweitens wurde deutlich, dass die konkrete Umsetzung der Leitideen und damit nicht nur die Ausgestaltung der Instrumente, sondern auch deren Implementierung als Ressource für soziale Solidarität zentral sind, denn „Kommunikation und Vertrauen sind […] Schlüsseldimensionen“ (Heidenreich 2022, S. 104). Diese Fragen hat die Wohlfahrtsstaatsforschung mit einigen Ausnahmen im Bereich Nichtleistungsbezug gerade erst begonnen zu adressieren (van Oorschot 1991). Mit der Wirkung von sozialpolitischen Instrumenten und Implementierungsprozessen auf persönliche Einstellungen, Werte und Zugehörigkeitsfragen ist eine Forschungslücke benannt, die es gemeinsam von der Wohlfahrtsstaats- und Organisationsforschung zu adressieren gilt.

## Footnotes

1 So Susanne Ferschl, DIE LINKE, am 13. März 2020 (BT-PP 19/153: 19035) und Bernd Rützel, SPD, am 22. April 2020 (BT-PP 19/155: 19249) in den Plenardebatten zur Bewältigung der durch das Virus ausgelösten Krise.

2 Kritiker:innen hingegen hat die Tatsache, dass Solidarität zum Gegenstand politischer Steuerungsbemühungen und Versprechen geworden ist, dazu veranlasst, die Möglichkeit, Solidarität institutionell zu erzwingen, infrage zu stellen. Zwang und Solidarität vertragen sich nicht, so das Argument. Der Zwang schwäche nicht nur die individuelle Freiheit (Habermas 1981, S. 531 f.), sondern auch die Selbsthilfepotenziale einer Gesellschaft.

3 Im Falle von Risiken wie Alter oder Krankheit, die weniger unter Selbstverschuldungsverdacht stehen, sind die Befragten eher zu Generalisierungsleistungen bereit als bspw. im Falle von Arbeitslosigkeit (Ullrich 2000, S. 207).

4 Wohlfahrtsstaatliche Akzeptanz wurde über die Zustimmung zu der Aussage „Der Staat muss dafür sorgen, dass man auch bei Krankheit, Not, Arbeitslosigkeit und im Alter ein gutes Auskommen hat.“ erhoben (Roller 2002).

5 Damit entfiel auch eine wichtige Funktion dieser Beziehungen, nämlich die Möglichkeit der individuellen Überprüfung von Bedürftigkeit und der gegenseitigen Kontrolle des Leistungsbezugs, was nun ebenfalls in den Bereich der rechtlich-formalen Organisation sozialer Sicherheit fiel.
